# [^18^F]FEDAC translocator protein positron emission tomography–computed tomography for early detection of mitochondrial dysfunction secondary to myocardial ischemia

**DOI:** 10.1007/s12149-021-01630-7

**Published:** 2021-06-03

**Authors:** Rui Luo, Lei Wang, Fei Ye, Yan-Rong Wang, Wei Fang, Ming-Rong Zhang, Feng Wang

**Affiliations:** 1 Department of Nuclear Medicine, Nanjing First Hospital, Nanjing Medical University, 68 Changle Road, Nanjing, 210006 China; 2 Department of Nuclear Medicine, Fuwai Hospital, National Center for Cardiovascular Diseases, Chinese Academy of Medical Sciences, Peking Union Medical College, Beijing, China; 3 Deparment of Cardiology, Nanjing First Hospital, Nanjing Medical University, Nanjing, China; 4 Department of Advanced Nuclear Medicine Sciences, National Institute of Radiological Sciences, National Institutes for Quantum and Radiological Science and Technology, 4-9-1 Anagawa, Inage-ku, Chiba, 263-8555 Japan

**Keywords:** FEDAC, ^18^F, TSPO, PET, Mitochondrial dysfunction, Myocardial injury

## Abstract

**Background:**

This study aimed to evaluate the biodistribution and kinetics of [^18^F]FEDAC targeting the translocator protein TSPO in the myocardium, and to explore its use for the identification of mitochondrial dysfunction. We also assessed the feasibility of [18F]FEDAC for the early detection of mitochondrial dysfunction associated with myocardial ischemia (MI).

**Methods:**

The radiochemical purity and stability of [^18^F]FEDAC were analyzed by radio-high-performance liquid chromatography (radio-HPLC). Its biodistribution and kinetics were evaluated by dissection and dynamic imaging using micro-positron emission tomography–computed tomography (micro-PET–CT) in healthy mice. [^18^F]FEDAC was also applied in an MI rat model and in sham-operated controls. Mitochondrial changes were observed by immunohistochemical staining and electron microscopy.

**Results:**

Radioactivity levels (%ID/g) in the myocardium in normal mice, determined by [^18^F]FEDAC, were 8.32 ± 0.80 at 5 min and 2.40 ± 0.10 at 60 min. PET showed significantly decreased uptake by injured cardiac tissue in MI rats, with maximal normal-to-ischemic uptake ratios of 10.47 ± 3.03 (1.5 min) and 3.92 ± 1.12 (27.5 min) (*P* = 0.025). Immunohistochemistry confirmed that TSPO expression was decreased in MI rats. Mitochondrial ultrastructure demonstrated significant swelling and permeability.

**Conclusion:**

[^18^F]FEDAC uptake is reduced in the injured myocardium, consistent with mitochondrial dysfunction. These results may provide new evidence to aid the early detection of mitochondrial dysfunction associated with myocardial ischemic injury.

## Introduction

Myocardial ischemia (MI) is a leading cause of cardiovascular mortality. Although the mortality of patients with coronary heart disease has declined in recent years due to evidence-based treatments and lifestyle changes, more than 7 million people worldwide still suffer from MI, associated with a massive economic impact [1, 2]. MI is mainly caused by the rupture of atherosclerotic plaques leading to thrombosis in the coronary artery lumen, which then obstructs the blood supply to the distal myocardium [3].

Imaging modalities including anatomical, functional, and molecular imaging are of great value in the diagnosis of cardiovascular diseases, allowing visualization and measurement of the underlying MI process [4]. Considerable effort has focused on the development of radionuclide imaging techniques for detecting MI in the cardiovascular system [5]. Mitochondria are crucial for regulating cell metabolism, producing ATP to promote cell growth, and participating in the apoptosis pathway, and mitochondrial dysfunction has been validated as a precursor of cell death [6–9]. The early detection of mitochondrial dysfunction thus presents a great clinical challenge in relation to mitochondrial dysfunction associated with reversible pathological changes, especially ultrastructural changes occurring during apoptosis and autophagy. Interventions at the stage of mitochondrial dysfunction may thus block cell death and allow the salvage of the ischemic myocardium, thus inhibiting myocardial remodeling and preserving global heart function. However, information on positron emission tomography (PET) tracers able to describe mitochondria dysfunction in the injured myocardium is currently lacking.

Myocardial injury causes mitochondrial swelling and the release of cytochrome c, which is involved in opening of mitochondrial permeability transition pores (mPTPs), modulated by the translocator protein TSPO [10–13]. mPTPs play a significant role in the generation of necrotic and apoptotic cell death [10, 11]. Downregulation of TSPO expression reduces the production of mitochondrial reactive oxygen species and limits oxidative stress [14]. Sequentially, the mitochondrial membrane potential is stabilized and mPTP opening is inhibited [14]. TSPO has been identified as an important component involved in the modulation of mitochondrial function [15–18], and PET using radiolabeled TSPO probes has been used for the non-invasive and reliable investigation of TSPO expression [19–21]. We previously synthesized an ^18^F-labeled PET probe, *N*-benzyl-*N*-methyl-2-[7,8-dihydro-7-(2-[^18^F]fluoroethyl)-8-oxo-2-phenyl-9H-purin-9-yl]acetamide ([^18^F]FEDAC), with high binding affinity for TSPO (Ki = 1.34 nM).

The aim of the current study was to evaluate the use of [^18^F]FEDAC PET–computed tomography (CT) for the detection of mitochondrial dysfunction associated with myocardial ischemia. In this work, we present results of studies with [^18^F]FEDAC in a rat model of coronary occlusion. These studies provide important data for assessing [^18^F]FEDAC as an potential effective imaging agent for detecting ischemic injuries in the heart.

## Materials and methods

### Radiosynthesis of [^18^F]FEDAC

Tosylate precursor solution (2 mg in anhydrous dimethyl sulfoxide 300 μL) was added to a reaction vessel containing dry [^18^F]F^−^, and the mixture was heated at 100–120 °C for 10–20 min. After purification by high-performance liquid chromatography (HPLC), 840–1520 MBq of [^18^F]FEDAC was obtained as an injectable solution at a beam current of 15 μA and proton bombardment of 15 min. The radiochemical purity and molar activity of the synthesized [^18^F]FEDAC were > 97% and 330–450 GBq/μmol, respectively.

### Blood half-life and biodistribution in Balb/c mice

All animal care and use procedures were performed strictly in accordance with the ethical guidelines and approval of the Nanjing Medical University, Animal Care and Use Committee. Healthy female Balb/c mice (7 weeks old, 20–22 g) were injected with [^18^F]FEDAC (3.7 MBq/100 μL) via the tail vein. Three mice from each group were sacrificed by cervical dislocation at various time points (1, 5, 15, 30, 60, 90 min). The main organs, including the adrenal gland, heart, liver, spleen, lung, kidney, stomach, and bone, were removed quickly and weighed, and blood samples were collected. The radioactivity levels in the tissues were measured using a 2480 γ-counter (Perkin-Elmer, Waltham, MA, USA), and expressed as mean ± standard deviation of the percentage of the injected dose per gram of tissue (%ID/g). All radioactivity measurements were corrected for decay.

### Coronary occlusion rat model

Wistar rats (male, 200–250 g) were anesthetized, intubated, and mechanically ventilated. Thoracotomy was performed via the fourth intercostal space, the heart was exposed, and an electrocardiographic monitor was connected. A 6–0 polypropylene suture (Ethicon, Somerville, NJ, USA) was then passed loosely around the left anterior descending coronary artery near its origin. Once the hemodynamics were stabilized, the left anterior descending coronary artery was occluded by tightening the suture loop for 30 min. Acute MI was considered successful based on regional cyanosis of the myocardial surface distal to the suture, accompanied by elevated ST segment on electrocardiography. The loop was then loosened and reperfusion was confirmed by return of the original color [22].

### Micro-PET–CT imaging

MI model (*n* = 5) and normal rats (*n* = 5) were divided into two groups. At 60 min after reperfusion, [^18^F]FEDAC (17 ± 2 MBq, 310–390 GBq/μmol) was injected via the tail vein. Micro-PET–CT images were then acquired using an Inveon small-animal PET scanner (Siemens, Knoxville, TN, USA), which provides 159 trans-axial slices 0.796 mM (center-to-center) apart, and 10-cm trans-axial and 12.7-cm axial fields of view. Before the scans, the rats were imaged in a prone position and anesthetized with 5% (v/v) isoflurane, maintained by 1–2% (v/v) isoflurane. After CT positioning and attenuation correction, emission scans were performed for 30 min after intravenous injection of [^18^F]FEDAC. PET images were obtained by summing the uptake between 0 and 30 min and reconstructed using ASIPro VMTM software, Analysis Tools and System Setup/Diagnostics Tool (Siemens Medical Solutions). Radioactivity was decay-corrected for injection time and expressed as the standardized uptake value (SUV), normalized for injected radioactivity and body weight. SUV was calculated as (radioactivity per cm^3^ tissue/injected radioactivity) × g body weight. Representative cardiac tissue specimens from the ischemic injury core and periphery were subsequently subjected to histological and immunohistochemical analyses, including autoradiography, hematoxylin and eosin (HE) staining, immunofluorescence, and transmission electron microscopy (TEM).

### In vitro autoradiography

MI model rats were sacrificed under ether anesthesia. Representative cardiac tissue specimens were harvested immediately, embedded in optimal cutting temperature compound (Sakura Finetek USA, Torrance, CA, USA), and frozen in hexane (Wako Pure Chemical Industries, Osaka, Japan). The sections (5 μm) were prepared using a cryotome, MICROM HM560 (Carl Zeiss, Jena, Germany), at a temperature of − 20 °C and mounted on adhesive silane-coated glass slides (Matsunami Glass Industries, Kyoto, Japan). In accordance with the established procedure [21], the sections were then pre-incubated in 50 mM Tris buffer (pH 7.4) at room temperature for 20 min followed by incubation in the same buffer containing [^18^F]FEDAC (18 MBq/L) at room temperature for 30 min. After incubation, the sections were washed twice for 2 min each in 50 mM cold fresh Tris–HCl buffer and for 10 s in distilled water. They were then dried with a warm air current, placed in contact with an imaging plate (BAS-MS 2325; Fujifilm, Tokyo, Japan) for 60 min, and analyzed using a Bio Imaging Analyzer System (BAS 5000; Fujifilm).

### Histological and immunohistochemical analyses

Representative cardiac tissue specimens were harvested from the ischemic injury core and periphery for analysis. Tissues were stained with HE and 2,3,5-triphenyltetrazolium chloride (TTC) to assess tissue damage and inflammation. The cardiac tissue sections were then assessed using immunofluorescence by staining with rabbit-anti TSPO/PBR (1:200; NP155) [23], followed by incubation with Alexa Fluor^®^ 488 goat anti-rabbit IgG (1:500; Invitrogen, Carlsbad, CA).

### Ultrastructural morphology analysis by TEM

Cell ultrastructure was observed by TEM (JEM-1010, Jeol Korea Ltd., South Korea). Briefly, cardiac tissues were fixed with 2.5% glutaraldehyde at 4 °C, dehydrated with graded ice-cold ethanols (70%, 80%, 90%, and 100%), and embedded in epoxy resin (EPON 812, Serva Feinbiochemica Heidelberg, Corporation. New York, USA). The blocks were cut into ultrathin sections using an ultramicrotome and stained with uranyl acetate/lead citrate.

### Statistical analysis

SPSS 19.0 software (SPSS Inc, Chicago, IL) was utilized for statistical analysis. All data were expressed as mean percentage ± standard deviation. Results were compared using Student’s *t* tests. Differences with a two-sided *P* value < 0.05 were considered significant.

## Results

### Radiolabeling and quality control

**Fig. 1:**
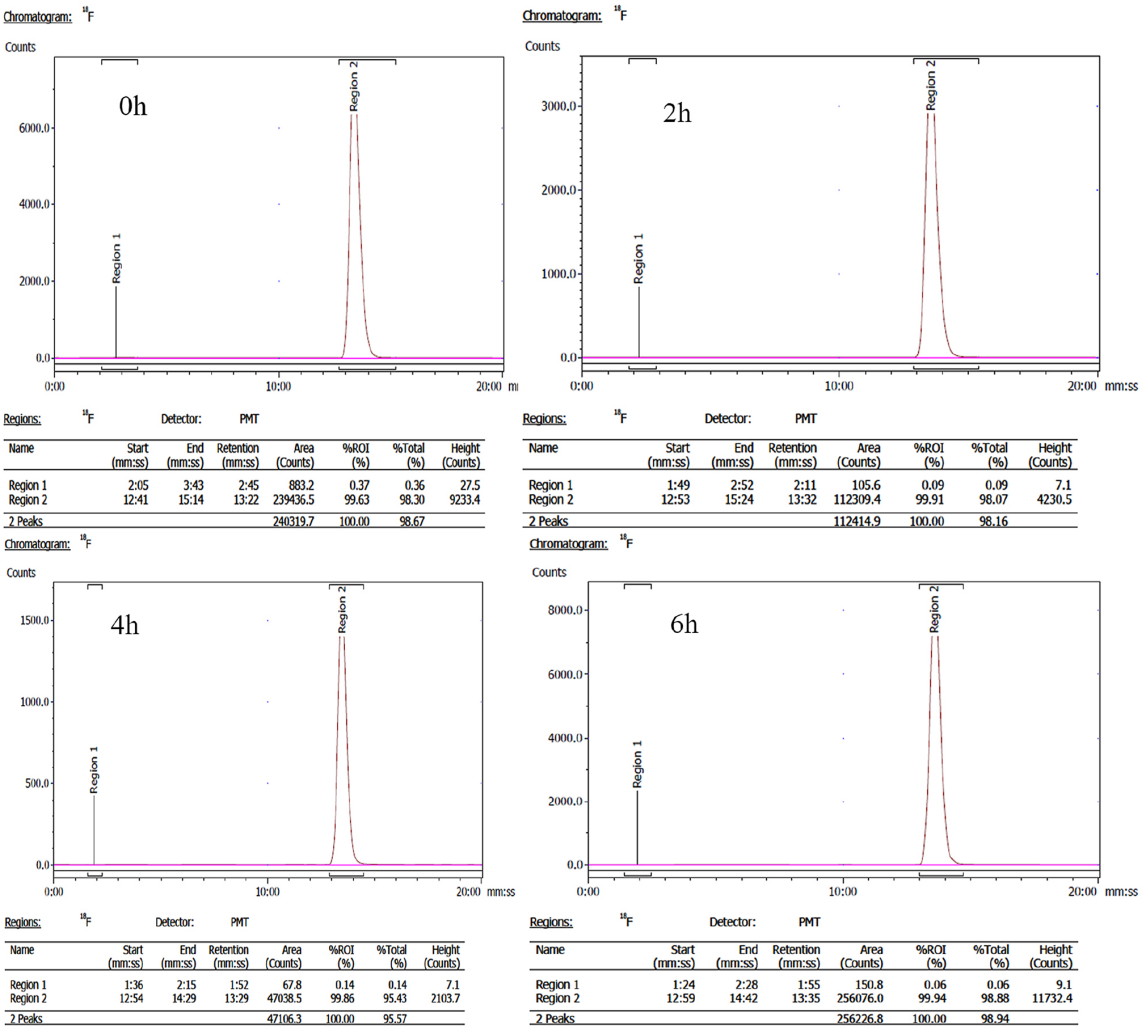
Radiochemical purity of [^18^F]FEDAC was > 90%, with a retention time of 13.32 min. [^18^F]FEDAC was stable at 6 h after radionuclide labeling, with no significant dissociation

The radiochemical purity of [^18^F]FEDAC determined by radio-HPLC was > 90%, with a retention time of 13.32 min. [^18^F]FEDAC was stable at 6 h after radionuclide labeling, with no significant dissociation (Fig. 1).

### Biodistribution and clearance kinetics in Balb/c mice

**Fig. 2:**
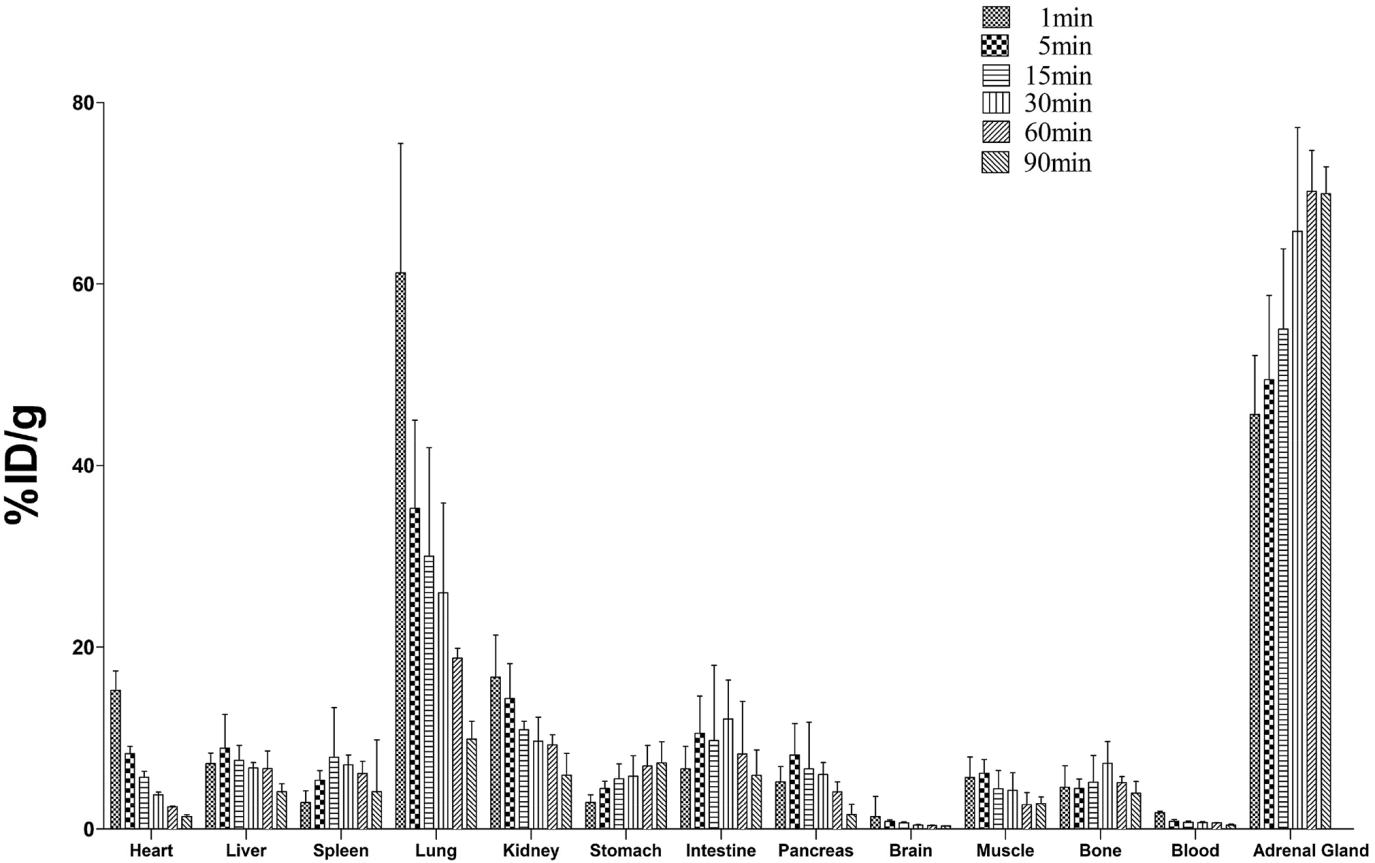
Biodistribution of [^18^F]FEDAC in the main organs in Balb/c mice. Most uptake occurred in the kidney, heart, and adrenal glands, and gradually decreased with time

[^18^F]FEDAC uptake in the main organs at various time points after intravenous injection is shown in Fig. 2. A high uptake of radioactivity (% ID/g) occurred immediately after 1 min and peaked in the blood, heart, and lung. Radioactivity levels in the lung reached 61.2 ± 14.2% ID/g, and then decreased rapidly within 5 min to 35.3 ± 9.7% ID/g. Radioactivity levels in the adrenal glands continued to increase up to 90 min. Comparatively lower uptakes were observed in the liver and muscle. Radioactivity uptake in the myocardium, as the targeted tissue in this study, was 8.32 ± 0.80% ID/g at 5 min, and decreased to 2.40 ± 0.10% ID/g at 60 min after injection. There was no significant increase in radioactivity levels in the bone in this experiment.

**Fig. 3:**
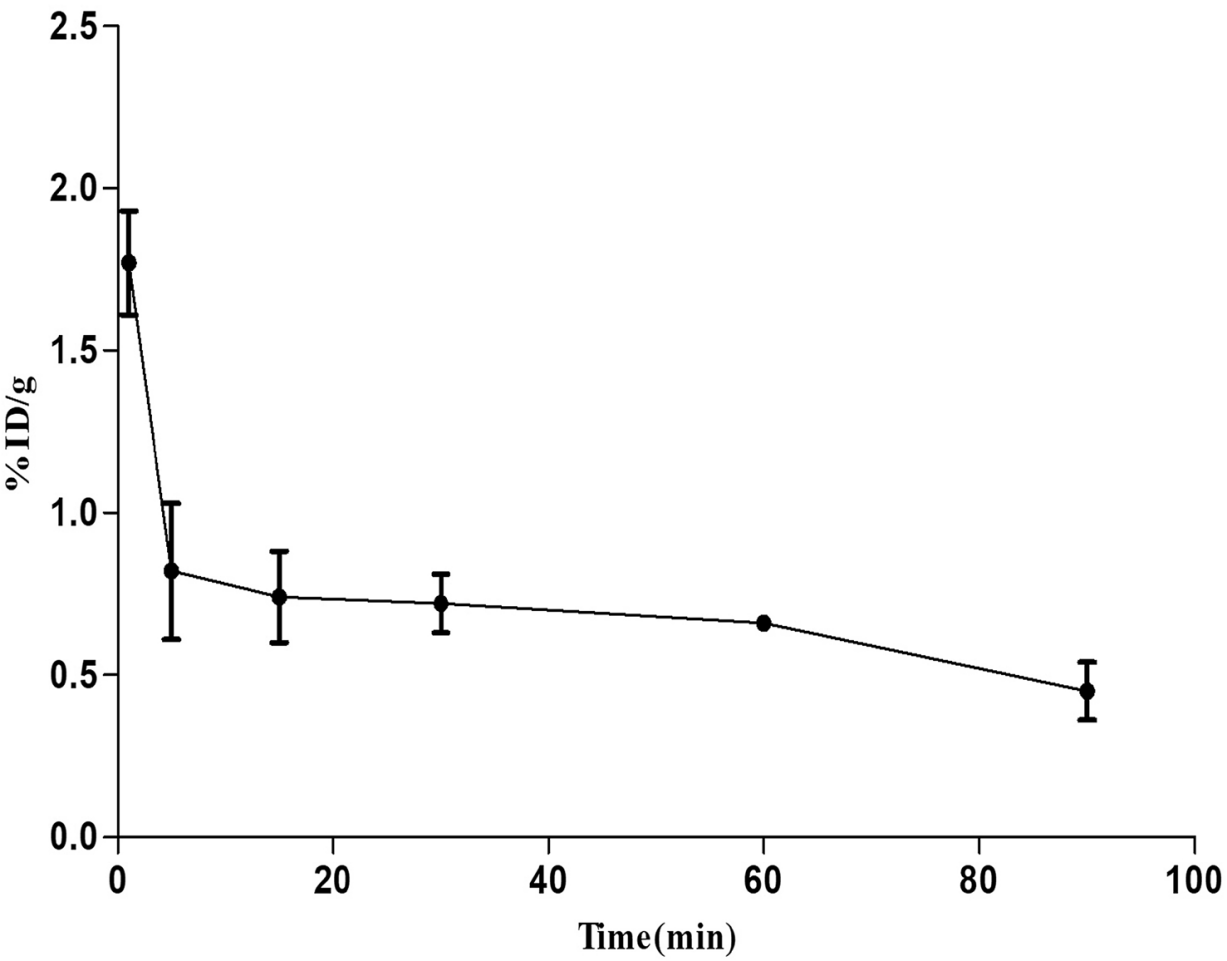
Two-compartment model of blood clearance–time profiles for [^18^F]FEDAC. The half-lives for the fast-clearance phase (α-phase) and slow clearance phase (β-phase) were 3.60 ± 0.2 and 108.28 ± 21.60 min, respectively. Radiotracer uptake in the blood at 90 min post-injection was 0.45 ± 0.09 (ID%/g)

A two-compartment model best described the blood clearance–time profiles for [^18^F]FEDAC in Balb/c mice. The half-life for the fast-clearance phase (α-phase) was estimated to be 3.60 ± 0.2 min (*n* = 3), whereas that of the slow clearance phase (β-phase) was 108.28 ± 21.60 min. Radiotracer uptake in the blood at 90 min post-injection was 0.45 ± 0.09 ID%/g (Fig. 3).

### Imaging myocardial injury in the rat model

**Fig. 4:**
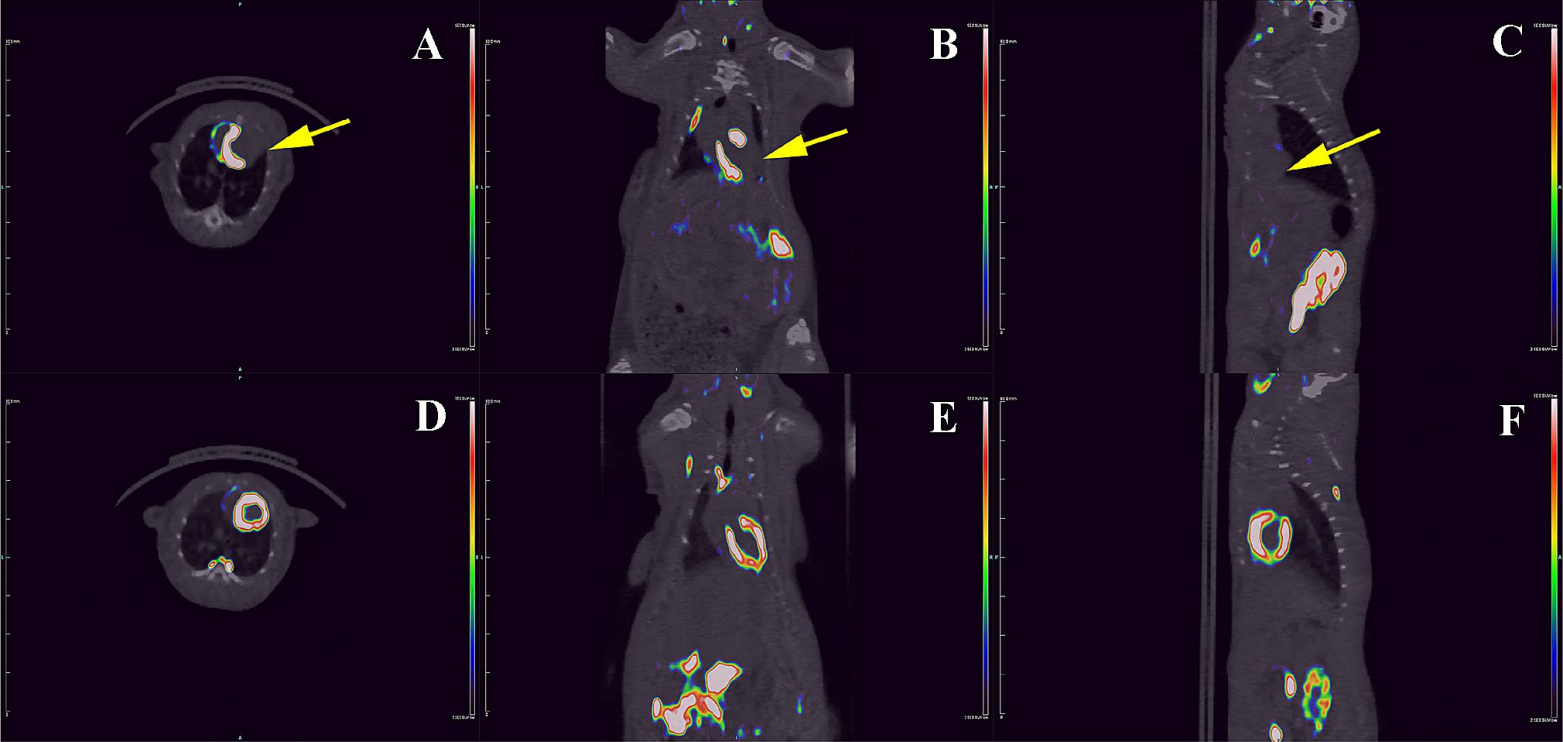
[^18^F]FEDAC micro-PET–CT for the detection of myocardial injury in a rat model. Decreased uptake or defects were visualized in the ischemic regions (**A**–**C**), with no significant decrease in uptake in the controls (**D**–**F**)

**Fig. 5:**
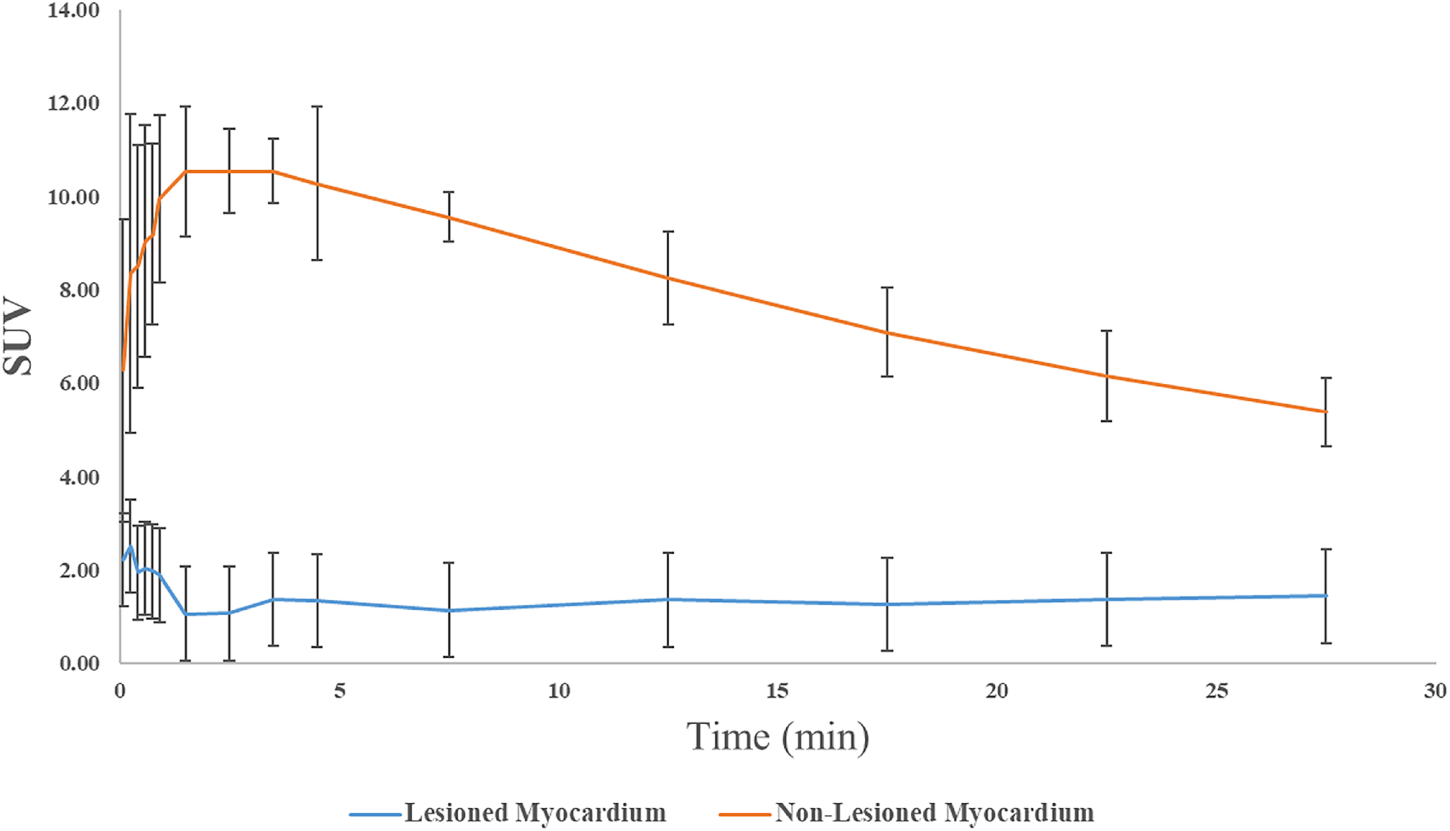
Time-activity and blood-clearance curves of [^18^F]FEDAC in a myocardial injury rat model. Maximal normal-to-ischemic uptake ratios were 10.47 ± 3.03 and 3.92 ± 1.12 (*P* = 0.025) at 1.5 and 27.5 min post-injection, respectively

Representative micro-PET–CT images of [^18^F]FEDAC uptake in MI model rats are shown in Fig. 4. PET images revealed a remarkable defect area at the ischemic myocardium in the anterior wall and the vertex cordis (Fig. 4). Signals from the blood pool in the heart chambers were minimal. The maximal normal-to-ischemic uptake ratios in the MI model were 10.47 ± 3.03 and 3.92 ± 1.12 (*P* = 0.025) at 1.5 min and 27.5 min, respectively (Fig. 5). Over the same period, the heart to liver ratio remained relatively ideal and stable (from 2.03 ± 0.42 to 2.24 ± 0.29 (*P* = 0.517), with no significant change over time. There were no adverse effects associated with the injected radiopharmaceutical.

### Autoradiography and TTC staining of ischemic rat hearts

**Fig. 6:**
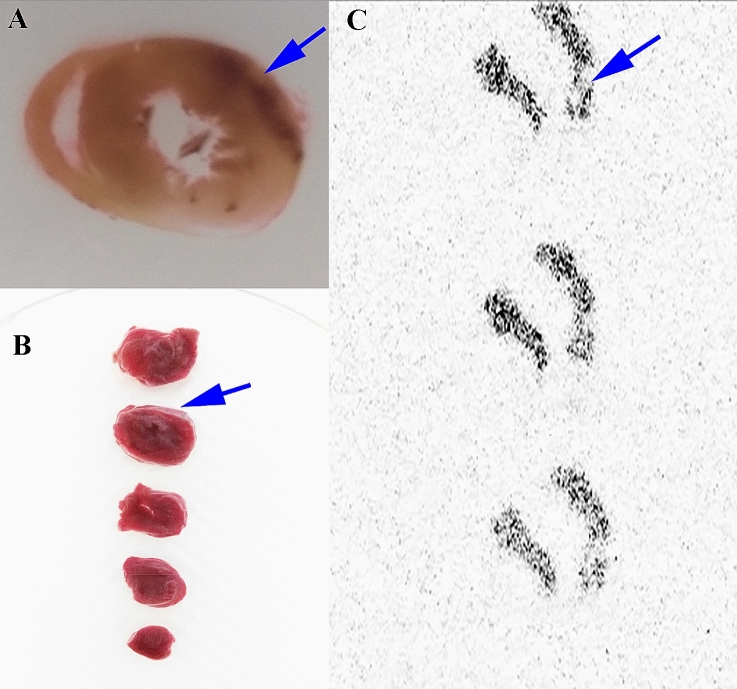
[^18^F]FEDAC in a myocardial injury rat model. **A** Frozen sections of myocardial tissue showing ischemic injury foci on the anterior wall of the left ventricle, consistent with the imaging sites. **B** TTC staining showing myocardial ischemia in the same area of the anterior wall of the left ventricle. **C** In vitro autoradiography results showing lower ^18^F-ligand binding in the injured compared with the normal area

Following PET evaluation, we visualized decreased levels of TSPO expression in the ischemic myocardium in rats using [^18^F]FEDAC. Surgery resulted in ischemic injury to the anterior wall and vertex cordis in the heart. Frozen sections of myocardial tissue revealed ischemic foci on the anterior wall of the left ventricle, consistent with the imaging sites (Fig. 6A). TTC staining showed MI damage in the same area of the anterior wall of the left ventricle (Fig. 6B). The in vitro autoradiography results are shown in Fig. 6C. [^18^F]FEDAC binding was lower in the injured compared with the normal area.

### Histopathological analysis

**Fig. 7:**
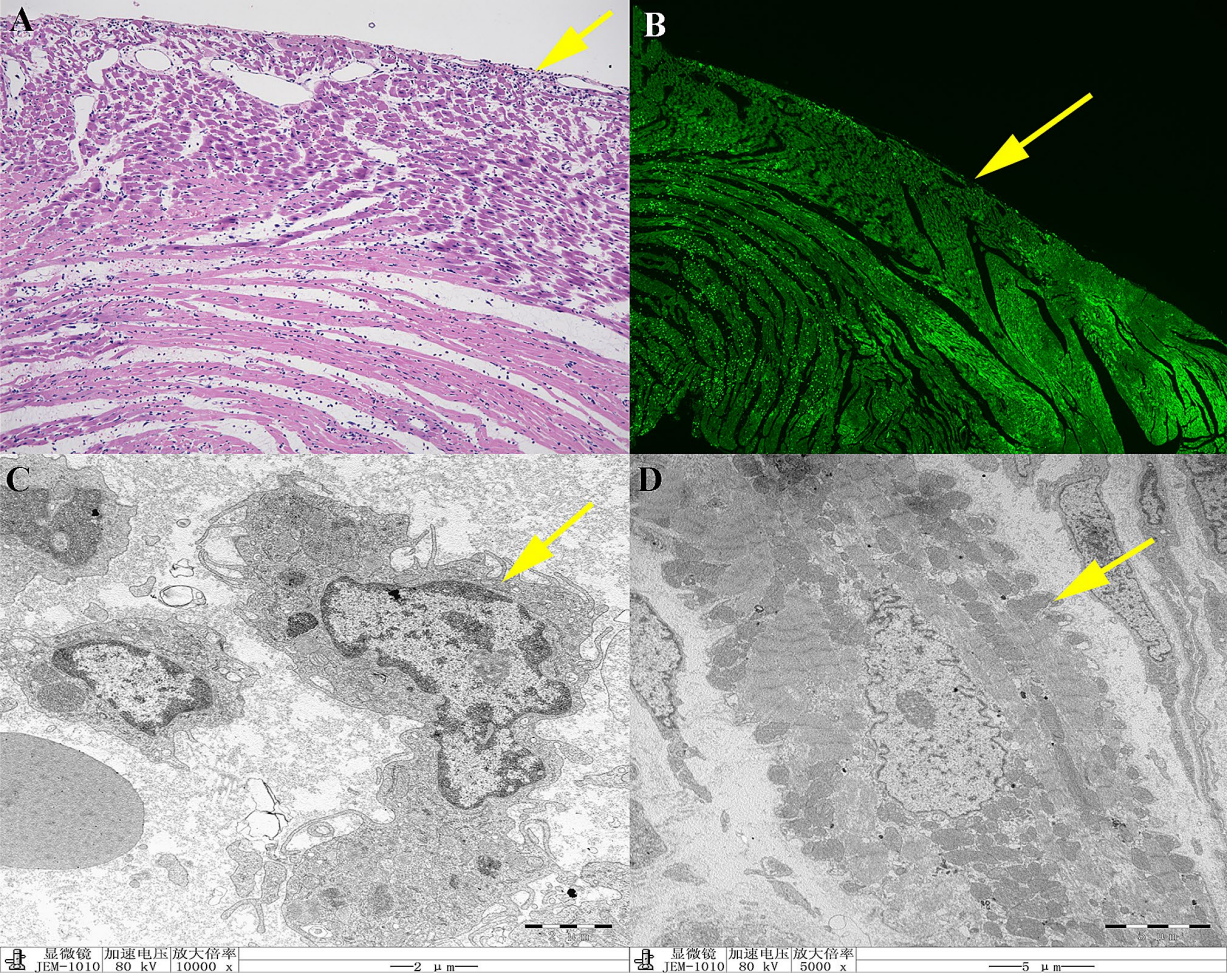
Decreased TSPO expression in the ischemic injured myocardium in a rat model was validated by histopathology, ultrastructure, and immunofluorescence, respectively. **A** HE staining showing nuclear fragmentation, myofibrillar degeneration, and local bleeding. **B** TSPO immunofluorescence showing low expression of TSPO in cardiac lesions compared with normal cardiomyocytes. **C**, **D** Transmission electron microscopy showing typical apoptotic and necrotic changes in ischemic injured myocardium, including cytoplasmic contraction, nuclear concentration and marginalization, and mitochondrial swelling

Representative histological images are shown in Fig. 7. HE staining showed fragmented nuclei, myofibril degeneration, and local hemorrhage, as typical changes associated with ischemic injury (Fig. 7A).

TSPO immunostaining of cardiac tissue sections is shown in Fig. 7B. TSPO expression was reduced in cardiac lesion sites in the myocardium compared with adjacent normal cardiac tissues. Ultrastructural changes associated with ischemic injury detected by TEM showed characteristic apoptotic and necrotic changes, including cytoplasm shrinkage, nuclear condensation and marginalization, and mitochondrial swelling (Fig. 7C, D).

## Discussion

Myocardial perfusion imaging has been well documented for the detection of MI and for risk stratification and prognosis evaluation in clinical practice. However, the relative spatial resolution of single-photon emission computed tomography systems, attenuation correction, and suboptimal distribution have limited the diagnostic efficacy for MI. CT angiography has been well validated for the detection of coronary stenosis and characterization of plaques, and invasive angiography is valuable for the detection of coronary artery stenosis, thus guiding treatment strategies, especially in cases of acute cardiovascular disease. However, all these diagnostic modalities have limited value for the detection of microcirculatory disturbances and coronary artery spasm. CT angiography, coronary fractional flow reserve, and myocardial perfusion imaging are the main clinical markers indirectly reflecting MI. Detection of MI by late gadolinium-enhanced cardiac magnetic resonance has shown promise, but lacks specificity and is easily affected by inflammation, edema, and other lesions. Attempts have, therefore, been made to identify novel biomarkers based on the pathophysiological mechanisms involved in myocardial injury progression, to identify new specific targets for the detection of ischemic myocardium.

[^11^C]PK11195 was the first PET ligand used for clinical imaging of TSPO. However, [^11^C]PK11195 had high non-specific binding and lipophilicity, resulting in the underestimation of TSPO expression and reduced sensitivity [24]. [^18^F]FEDAC together with PET–CT have been utilized for the detection of neuroinflammation, nonalcoholic fatty liver disease, and liver fibrosis in preclinical studies [25–28]. [^18^F]FEDAC was developed as a specific radioligand for TSPO imaging [25], prompting the current investigations in myocardial injury models. [^18^F]FEDAC has demonstrated great potential as a PET probe due to its relatively simple synthesis and high degree of safety in non-clinical tests [29]. Furthermore, its favorable biodistribution, especially its predominant clearance from the kidney and low physiological uptake in the liver, guaranteed high-quality images.

Herein, we evaluated the use of [^18^F]FEDAC PET–CT for visualizing the involvement of TSPO in the modulation of mitochondrial dysfunction in myocardial injury models. [^18^F]FEDAC had a purity > 90% and chemical and radiochemical stability, with no dissociated radioactivity upon radiolabeling. The current results verified the favorable biodistribution and enhanced pharmacokinetic profile of [^18^F]FEDAC in a myocardial injury model in vivo. Its characteristics, such as rapid blood clearance and low hepatic background, were particularly notable. Furthermore, a two-compartment model best represented the blood clearance–time profiles of [^18^F]FEDAC, with a half-life for the fast-clearance phase (α-phase) of 3.60 ± 0.20 min. These results indicated that [^18^F]FEDAC was suitable for the detection of myocardial injury, due to its better distribution.

[^18^F]FEDAC PET–CT revealed that TSPO expression was pathologically decreased in ischemic myocardium compared with normal cardiac tissue. Differences in TSPO expression in the MI model could be detected and quantified with [^18^F]FEDAC PET–CT, with normal-to-ischemic uptake ratios from 10.47 ± 3.03 (1.5 min) to 3.92 ± 1.12 (27.5 min) (*P* = 0.025), enabling the non-invasive imaging of myocardial injury in the acute phase as early as 30 min after injection. In vitro autoradiography further supported the specific binding of [^18^F]FEDAC to TSPO in MI cardiac sections.

Mitochondrial dysfunction plays a key role in the pathogenesis of MI and reperfusion injury [10–13]. Mitochondrial swelling, abnormalities of mitochondrial complex-1 in the electron transport chain, the release of cytochrome c, mPTP opening, and necrosis and apoptosis of cardiomyocytes have been described in patients with MI [16, 30]. Histopathological staining, including HE, TTC, and immunofluorescence staining, and ultrastructural examination are widely used to detect apoptosis. Large numbers of apoptotic cells, characterized by mitochondrial swelling, cell shrinkage, condensed nuclei, and the appearance of apoptotic bodies, were observed in the cardiac tissue specimens, and these results were confirmed by TEM. These observations were in accord with the low expression of TSPO in injured cardiac tissue. The decreased uptake of [^18^F]FEDAC in the PET images was consistent with the observed histopathological features, indicating that the ischemic myocardium showed lower levels of TSPO expression. [^18^F]FEDAC PET thus displayed good sensitivity and specificity for detecting pathological changes and for assessing the distribution of injured regions in MI rats.

^99m^Tc-Sestamibi and ^99m^Tc-Tetrofosmin imaging are current available tracers for myocardial perfusion imaging, but as previous study showed, the most predominant uptake of distribution are liver and intestines[31], increased liver, intestinal, or gastric activity may create a major problem in the visual and quantitative interpretation of the posterior basal segment of myocardium. [^18^F]FEDAC may aid the early detection of MI injury if combined with myocardial perfusion imaging, and may thus support early interventions and decrease myocardial remodeling in ischemic cardiovascular disease. We realize that there were limitations to our initial evaluation of TSPO imaging of myocardial injury. Radioactivity levels in the lung reached 61.2 ± 14.2% ID/g, and then decreased rapidly within 5 min to 35.3 ± 9.7% ID/g, Radioactivity levels in the adrenal glands continued to increase up to 90 min. Although the image quality was not affected due to the high radioactivity uptake of the myocardium, future studies are required to modify structure of FEDAC to improve the biological distribution.

Overall, the present study demonstrated that [^18^F]FEDAC uptake was significantly decreased in the ischemic myocardium, correlated with the apoptotic index (especially mitochondrial dysfunction), histological findings, ultrastructural changes, and autoradiography results. These results suggest the feasibility of using [^18^F]FEDAC PET–CT for the early detection of myocardial injury, and also shed further light on the use of molecular imaging for assessing mitochondrial dysfunction.

## Conclusions

In summary, [^18^F]FEDAC PET is a sensitive method for the detection of mitochondrial dysfunction associated with myocardial injury, which was shown to be consistent with decreased TSPO expression. TSPO might thus serve as a promising biomarker reflecting mitochondrial dysfunction in myocardial vascular disease. However, further studies are warranted to validate these preliminary findings.
